# Retinal Structural and Vascular Changes in Patients with Coronary Artery Disease: A Systematic Review and Meta-Analysis

**DOI:** 10.3390/life14040448

**Published:** 2024-03-28

**Authors:** Alexandra Cristina Rusu, Karin Ursula Horvath, Grigore Tinica, Raluca Ozana Chistol, Andra-Irina Bulgaru-Iliescu, Ecaterina Tomaziu Todosia, Klara Brînzaniuc

**Affiliations:** 1 Doctoral School of Medicine and Pharmacy, George Emil Palade University of Medicine, Pharmacy, Science, and Technology of Targu Mures, 540142 Targu Mures, Romania; alexandracristina.rusu@gmail.com; 2 Department of Ophthalmology, Emergency County Hospital Targu Mures, 540136 Targu Mures, Romania; 3 Faculty of Medicine, George Emil Palade University of Medicine, Pharmacy, Science, and Technology of Targu Mures, 540142 Targu Mures, Romania; klara.brinzaniuc@umfst.ro; 4 Faculty of Medicine, Grigore T. Popa University of Medicine and Pharmacy, 700115 Iasi, Romania; grigore.tinica@umfiasi.ro (G.T.); bulgaru-iliescu.andra-irina@email.umfiasi.ro (A.-I.B.-I.); ecaterinatomaziu@gmail.com (E.T.T.); 5 Prof. Dr. George I.M. Georgescu Cardiovascular Diseases Institute, 700503 Iasi, Romania

**Keywords:** coronary artery disease, retinal, microvascularization, meta-analysis, optical coherence tomography angiography

## Abstract

Background: Retinal microvascular anomalies have been identified in patients with cardiovascular conditions such as arterial hypertension, diabetes mellitus, and carotid artery disease. We conducted a systematic review and meta-analysis (PROSPERO registration number CRD42024506589) to explore the potential of retinal vasculature as a biomarker for diagnosis and monitoring of patients with coronary artery disease (CAD) through optical coherence tomography (OCT) and optical coherence tomography angiography (OCTA). Methods: We systematically examined original articles in the Pubmed, Embase, and Web of Science databases from their inception up to November 2023, comparing retinal microvascular features between patients with CAD and control groups. Studies were included if they reported sample mean with standard deviation or median with range and/or interquartile range (which were computed into mean and standard deviation). Review Manager 5.4 (The Cochrane Collaboration, 2020) software was used to calculate the pooled effect size with weighted mean difference and 95% confidence intervals (CI) by random-effects inverse variance method. Results: Eleven studies meeting the inclusion criteria were incorporated into the meta-analysis. The findings indicated a significant decrease in the retinal nerve fiber layer (WMD −3.11 [−6.06, −0.16]), subfoveal choroid (WMD −58.79 [−64.65, −52.93]), and overall retinal thickness (WMD −4.61 [−7.05, −2.17]) among patients with CAD compared to controls (*p* < 0.05). Furthermore, vascular macular density was notably lower in CAD patients, particularly in the superficial capillary plexus (foveal vessel density WMD −2.19 [−3.02, −1.135], *p* < 0.0001). Additionally, the foveal avascular zone area was statistically larger in CAD patients compared to the control group (WMD 52.73 [8.79, 96.67], *p* = 0.02). Heterogeneity was significant (I^2^ > 50%) for most features except for subfoveal choroid thickness, retina thickness, and superficial foveal vessel density. Conclusion: The current meta-analysis suggests that retinal vascularization could function as a noninvasive biomarker, providing additional insights beyond standard routine examinations for assessing dysfunction in coronary arteries.

## 1. Introduction

Today, ischemic heart disease remains a leading cause of death globally, being responsible for approximately 16% of the total mortality rate at any given time, according to the World Health Organization [1]. Although both modifiable and non-modifiable risk factors for coronary artery disease (CAD) are well known and understood [2], the significant disease burden fuels the drive for finding innovative approaches in early diagnosis and improved risk stratification.

The last two decades have brought novel screening and diagnostic non-invasive imaging methods into current medical practice, such as coronary computed tomography angiography (CCTA). Despite their availability, both invasive and non-invasive coronary angiography—recognized as gold-standard diagnostic methods for coronary artery disease (CAD)—have limited utility for widespread screening in the general population due to various factors, such as the need for intravenous iodinated contrast material injection, X-ray exposure, patient- and/or technique-related lower image quality, imaging artefacts, risk of local and systemic complications, and patient-related contraindications. Moreover, such investigative methods fail to diagnose dysfunction of the coronary microcirculation, the underlying cause of angina in almost 50% of cases, which is associated with unfavorable prognosis [3]. 

Coronary microcirculation status can be estimated using indirect methods such as the Thrombolysis in Myocardial Infarction (TIMI) myocardial perfusion grade during coronary angiography and the coronary flow reserve measured using positron emission tomography, magnetic resonance imaging (MRI), and CCTA [4]. Unfortunately, all these techniques are of limited use, being associated with increased examination costs and offering only a rough estimate of the microvascular status. Currently, there is an acute need for novel non-invasive methods that could be widely used for screening, risk assessment, and stratification in CAD. 

The relationship between retinal microvascular anomalies and systemic vascular disease has been recognized since the nineteenth century, when Gunn RM performed the first ophthalmoscopic studies and identified retinal microvascular anomalies in patients with general arterial disease, chronic renal disease, and increased arterial tension [5,6]. In 2001, Wong et al. performed an extensive review of retinal anomalies (directly or indirectly arteriolar) encountered in cardiovascular diseases such as hemorrhages, microaneurysms, macular oedema, exudates, retinal ischemia, arteriolar narrowing, and arteriovenous nicking [7]. Thus, retinal vessels could be considered a mirror of the coronary microvasculature, as they share the size and structure of coronary arterioles and capillaries. Despite early recognition, for more than a century, retina examination methods (ophthalmoscopy, fundus photography) did not offer objective, quantitative, automatic, and reproducible tools for assessing microvascular changes. 

Optical coherence tomography (OCT) and optical coherence tomography angiography (OCTA) are novel non-invasive methods that enable precise visualization and quantification of the retinal and choroidal vasculature, structures that were previously accessible for objective study only through the invasive usage of intravenous injections of contrast agents. Both OCT and OCTA have already proven their use in evaluating patients with cardiovascular diseases. The impact of arterial hypertension on choroidal thickness and retinal microvascularization has been investigated by OCT [8] and OCTA [9], validating the fact that hypertensive patients show reduced choroidal thickness and lower superficial and deep vascular density compared to healthy controls. In patients with chronic heart failure and reduced left ventricular ejection fraction (LVEF), Alnawaiseh et al. [10] identified reduced retinal and optic nerve head perfusion through OCTA. The authors further postulated that assessing retinal perfusion via OCTA could offer valuable insights into the overall microcirculation and hemodynamic condition of individuals with heart failure. 

In the case of CAD, several studies involving OCT and OCTA have reported decreased vascular density in the eyes of patients with coronary artery stenosis together with thinning of the retinal nerve fiber layer (RNFL), increased foveal avascular zone (FAZ) area, and other changes associated with increased CAD severity and risk of adverse events. Impaired endothelial cells with altered function have been noted as main determinants of both retinal microvascular anomalies (arteriolar narrowing, arteriovenous nicking, vessel tortuosity) and the progress of coronary atherosclerosis. This common ethiopathogenic factor could explain the link between decreased retinal vascular density and coronary stenotic lesions [11]. 

Considering this, we performed a comprehensive meta-analysis of studies analyzing retinal vascular changes using OCT and OCTA to provide more reliable evidence for the utilization of these methods in the diagnosis and monitoring of patients with CAD.

## 2. Materials and Methods

### 2.1. Literature Search Strategy

The international prospective register of systematic reviews (PROSPERO) registration number for this study is CRD42024506589. 

This investigation adhered to the guidelines outlined in the Preferred Reporting Items for Systematic Reviews and Meta-Analysis (PRISMA) [12]. Two authors (A.C.R., R.O.C.) independently searched the Pubmed, Embase, and World of Science (WOS) databases for cohort or cross-sectional studies examining the relationship between retinal microvascular changes and CAD using the following queries: [“coronary artery disease” OR “coronary heart disease”] AND [“optical coherence tomography angiography retina” OR “optical coherence tomography retina” OR “OCTA retina” OR “OCT angiography retina” OR “OCT retina”]. No language limitation was applied. Identified records were supplemented by manual searching of relevant references found in retrieved articles. 

### 2.2. Inclusion and Exclusion Criteria

Chosen studies met the following criteria: (1) the study included CAD patients with their disease confirmed by coronary angiography or CCTA and no history of preexisting quantifiable retinopathy (diabetic, hypertensive), ocular surgery, high myopia, dense cataracts or other macula-obscuring media opacities, glaucoma, retinal laser photocoagulation therapy, or systemic vasculopathy; (2) the study included individuals with no coronary lesions or significant ocular diseases as controls; (3) the study included cross-sectional observational research; (4) the main outcomes of the study included superficial capillary plexus (SCP) density, deep capillary plexus (DCP) density, retinal nerve fiber layer (RNFL) thickness, choroid thickness (CTh), radial peripapillary capillary (RPC) density, foveal avascular zone (FAZ) area, ganglion cells-inner plexiform layer (GC-IPL) thickness, and retina thickness. 

A study was excluded from the meta-analysis if it (1) provided different outcomes; (2) did not include a control group; (3) presented only the results of the statistical analysis with no baseline data; or (4) was a low-quality investigation. 

### 2.3. Data Extraction

Three investigators (K.U.H., R.O.C., A.I.B.I.) extracted the following data from each selected study: first author’s surname, publication year, study design, the country in which the study was conducted, sample size, OCT/OCTA device, baseline data, outcome data. A 4th investigator, K.B., analyzed the final data and referred to the original article in the case of a difference between extracted data. 

The Newcastle-Ottawa Scale (NOS) was used to evaluate the quality of the included studies. All studies scored 7 or 8 stars (high-quality studies) (Table 1). Two investigators independently evaluated selected articles (A.C.R., E.T.T.).

### 2.4. Statistical Analysis

Studies included in the analysis were functionally identical (cross-sectional), the effect size differing mainly because of sampling. For statistical analysis, we have applied the same methodology as in a previously published study [23]. We used Review Manager (RevMan) 5.4 (The Cochrane Collaboration, 2020) software to calculate the pooled effect size with weighted mean difference (WMD) and 95% confidence intervals (CI) by inverse variance method according to the recommendations of the *Cochrane Handbook of Systematic Reviews* [24]. When median, range, and/or interquartile range were reported in the studies together with the sample size, mean and standard deviation were estimated by following the methods described by Luo et al. [25] and Wan et al. [26] in order to be used for the pooled analysis. When mean was reported together with 95% CI, RevMan Calculator (Cochrane Training) allowed us to compute standard deviation. 

The heterogeneity among studies was estimated by chi-squared-based Q test and I^2^ statistics; a *p* value > 0.05 for the Q test and an I^2^ > 50% were considered measures of important heterogeneity. In the case of low heterogeneity, a fixed model effect was used for the meta-analysis compared to the random effect method with increased heterogeneity. When needed, online supplements were consulted, and the authors were contacted to provide additional data. Publication bias was assessed by analyzing the asymmetry of the funnel plot.

## 3. Results

**Figure 1:**
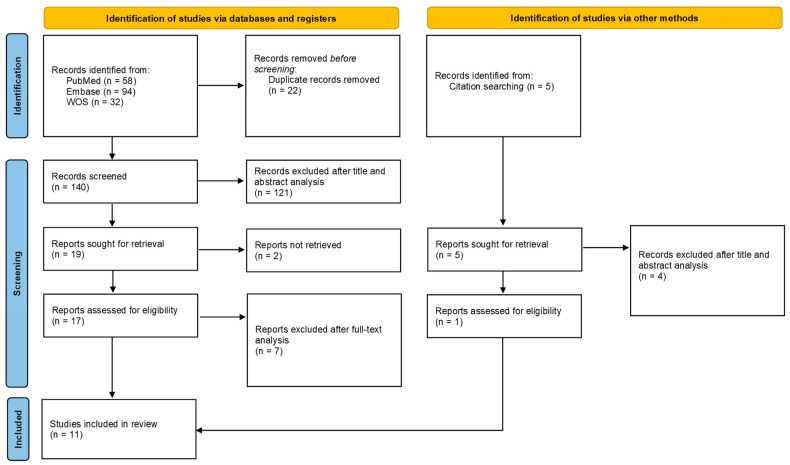
PRISMA flow chart of the selection process.

The literature search yielded the following number of records: Pubmed, 220; Embase, 171; WOS, 32. Identified references were checked for duplicates, and a total of 140 records resulted. After manually screening the abstracts, a further 121 reviews, conference abstracts, short communications, or original articles containing limited or no actual data were removed. A total of 19 abstracts qualified for further evaluation of the full-text article, to decide whether inclusion and exclusion criteria were met. Another nine articles were excluded during this step due to missing data, absence of a control group, or if full-text retrieval proved impossible. In the end, 11 studies were finally included in the quantitative synthesis (10 from the initial search, 1 identified through citation searching after full-text analysis). The results of the selection process are detailed in Figure 1, and the summary of included studies is in Table 2. 

The 11 selected studies included a total of 1536 CAD patients and 925 control patients. The baseline characteristics of patients from the studied groups are reported in Table 3.

Mean age difference between the CAD and no-CAD groups was less than one year (age-matched CAD and no-CAD groups across analyzed studies) in favor of older individuals with CAD, registering thusly a higher incidence of CAD risk factors compared to the control group. 

**Figure 2:**
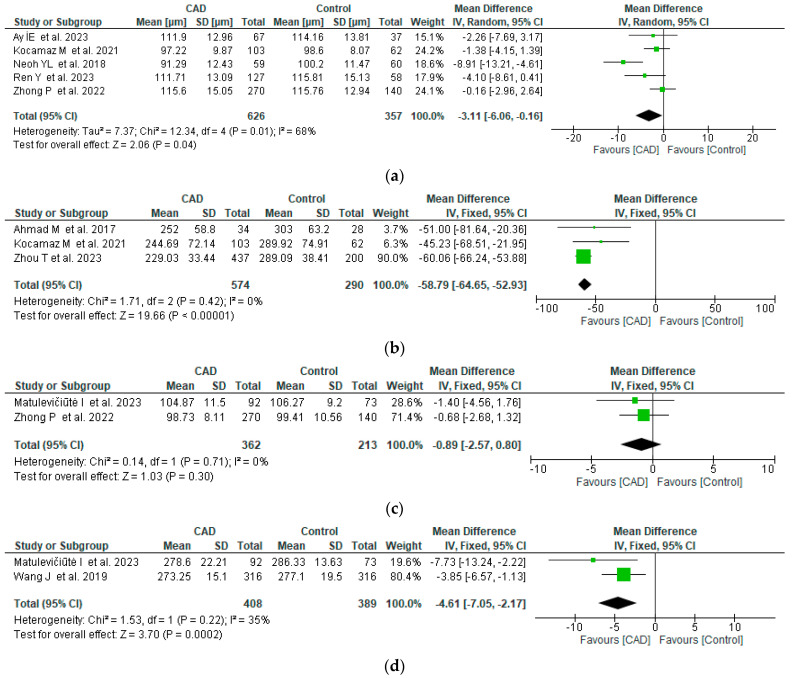
Forest plot of retinal layers thickness between CAD and control groups [11,13,14,15,16,18,19,20,22]. (**a**) RNFL thickness, (**b**) subfoveal choroid thickness, (**c**) ganglion cell-inner plexiform layer thickness, (**d**) retina thickness.

Five studies analyzed retinal nerve fiber layer thickness (μm) in patients with CAD. The pooled WMD was −3.11 (95% CI: −6.06 to −0.16, *p* = 0.04), with moderate heterogeneity, revealing that RNFL thickness is lower in CAD patients compared to controls. Subfoveal choroid thickness (μm) was quantified in three studies and was also significantly lower in CAD patients, with a pooled WMD of −58.79 (95% CI: −64.45 to −52.93, *p* = 0.0003) and no heterogeneity across studies. Ganglion cell-inner plexiform layer and total retina thickness were only quantified by two studies, each with low heterogeneity. In the case of the GC-IPL thickness, the difference between CAD and controls was not statistically significant (*p* = 0.3), but the total retinal thickness was significantly lower in CAD (pooled WMD −4.61 μm, 95% CI: −7.05 to −2.17, *p* = 0.0002) (Figure 2).

**Figure 3:**
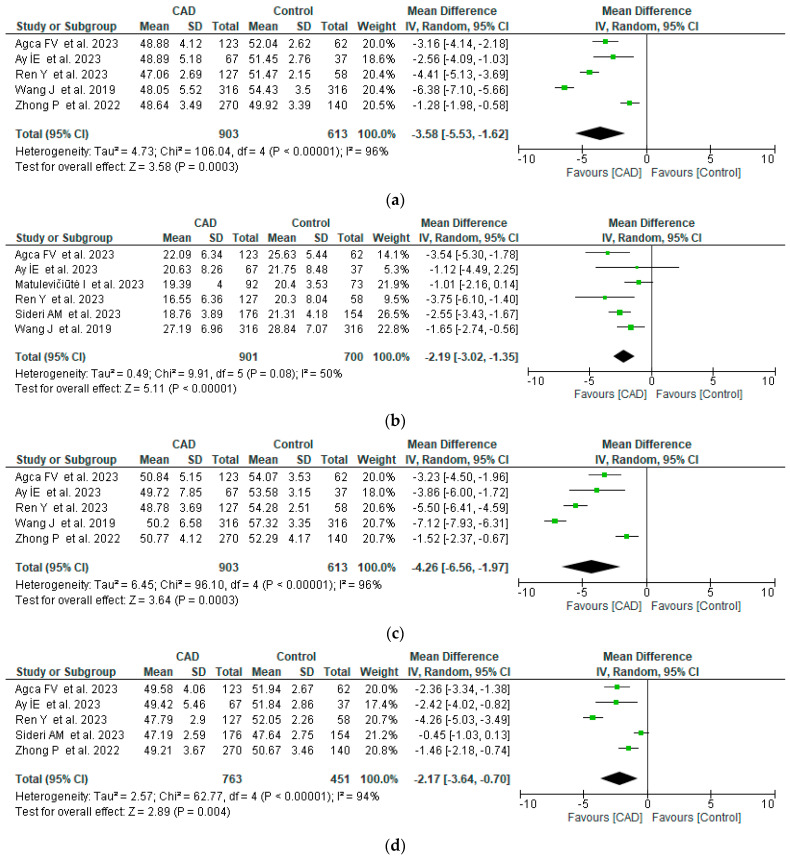
Forest plots of SCP vessel density between CAD and control groups [11,15,17,18,19,20,21]. (**a**) Whole vessel density, (**b**) fovea vessel density, (**c**) parafovea vessel density, (**d**) perifovea vessel density.

Retinal capillary plexus density (superficial and deep) in the macula was analyzed for the whole image and for specific regions from the center to the periphery (fovea, parafovea, and perifovea rings). All retinal microvascular structures were severely damaged in CAD patients compared to controls. Vascular densities in the macula were significantly lower in patients with CAD (Figure 3) for all regions of the macula, especially in the superficial capillary plexus. Heterogeneity across the studies was moderate or considerable because of differences between studied populations (all races), CAD severity, associated diseases, and devices used for quantification, but all studies converged in measuring lower vascular densities in CAD patients. The en face superficial vessel density (%) was between −6.38 and −1.28 lower than controls, the fovea superficial vessel density between –3.75 and −1.01 lower, the parafovea superficial vessel density between −7.12 and −1.52 lower, and the perifovea superficial vessel density between −4.26 and −0.45 lower than controls.

**Figure 4:**
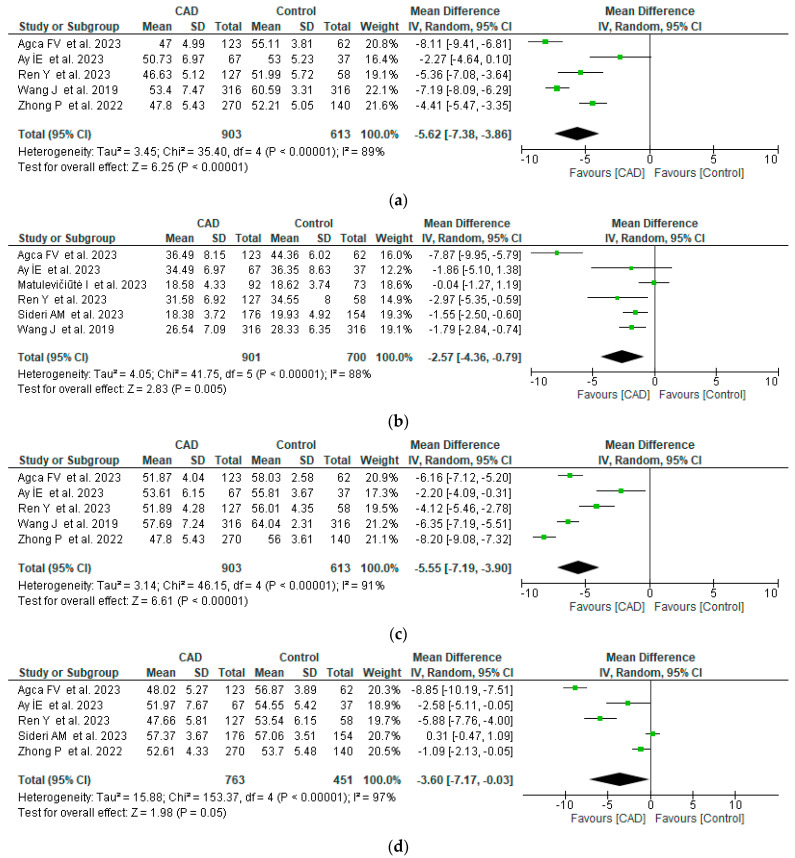
Forest plots of DCP vessel density between CAD and control groups [11,15,17,18,19,20,21]. (**a**) Whole vessel density, (**b**) fovea vessel density, (**c**) parafovea vessel density, (**d**) perifovea vessel density.

The deep capillary plexus density was also affected, with values ranging between −8.11 and −2.27 for the whole image, −7.87 and −0.04 for the fovea, −8.20 and −2.2 for the parafovea, and −8.85 and 0.31 for the perifovea, with considerable heterogeneity (Figure 4).

**Figure 5:**
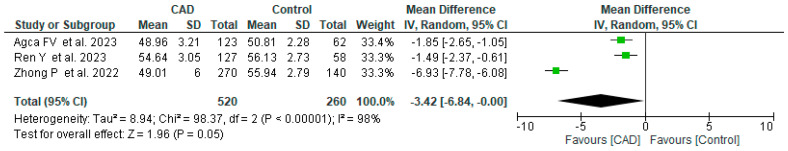
Forest plot of RPC whole vessel density between CAD and control groups [11,17,20].

The radial peripapillary capillary vessel density (%) was quantified by three studies that also converged in measuring lower values for CAD patients (−6.93 to −1.49 lower, pooled WMD −3.42, *p* = 0.05), with increased heterogeneity (Figure 5).

**Figure 6:**
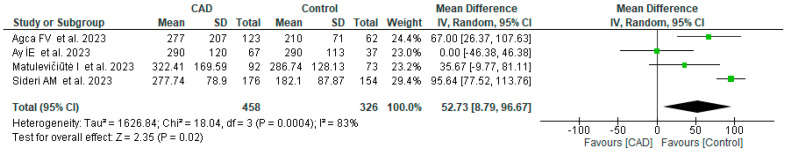
Forest plot of FAZ area between CAD and control groups [17,18,19,21].

In the case of the foveal avascular zone (FAZ) area (μm^2^), pooled WMD indicated a 52.73 μm^2^ higher area in CAD patients compared to controls (*p* = 0.0003), with considerable heterogeneity across the four studies quantifying this feature. A single study reported a non-significant difference in FAZ area between CAD and no-CAD patients, but it included a limited number of cases (67 CAD patients, 37 healthy controls) compared to other research (Figure 6). 

## 4. Discussion

To the best of our knowledge, this meta-analysis represents the first attempt to assess and compare retinal structural and vascular changes using OCT and OCTA between individuals with coronary artery disease (CAD) and a control group. We identified eleven relevant studies and aggregated various potentially valuable parameters for screening and grading CAD severity, including macular superficial and deep vascular density (whole, foveal, parafoveal, perifoveal), FAZ area, and retinal layers’ thickness. 

Despite moderate to substantial heterogeneity across the eligible studies, the combined data revealed a significant decrease in macular superficial and vascular density, a notable thinning of retinal layers, and a significant increase in FAZ area in CAD patients compared to controls. Retinal structural alterations and vascular changes are intricately connected. The blood supply for SCP comes from the central retinal artery, whereas the DCP receives its nutritional support also from the choriocapillaris. The FAZ lacks vascular structures, being oxygenated by neighboring choroidal tissue supplied by the posterior ciliary artery. Additionally, the optic nerve head is nourished by the radial peripapillary capillaries [27]. Consequently, the thickness of retinal layers is influenced by retinal vascularization and the obstruction and constriction of various vascular sources, leading to loss of perfusion in DCP and SCP, with subsequent thinning of retinal layers. 

In 2012, Machalińska et al. demonstrated endothelial dysfunction as a key feature in the development of both systemic atherosclerosis and age-related macular degeneration. This finding suggests a direct connection between retinal vessels and overall atherosclerosis, including CAD [28]. Both the retina and coronary arteries are affected by endothelial dysfunction and disruptions in the autoregulatory system of the vessels. Impaired coronary autoregulation is linked to long-term fatal events in stable CAD [29]. The retinal and coronary microvasculature exhibit parallel responses to prevalent cardiovascular risk factors such as arterial hypertension, diabetes mellitus, hypercholesterolemia, and obesity. This response involves endothelial disruption and reduced production of nitric oxide (NO), accelerating the inflammatory process and resulting in compromised angiogenesis [30].

Compared to coronary arteries, the retina can be easily assessed using non-invasive methods due to its location and structure. In recent years, OCT and OCTA, state-of-the-art non-invasive imaging methods, have become widely accessible for an accurate, objective, and reproducible evaluation of the retinal structure and vascularization. Prior methods were subjective (ophthalmoscopy) or more patient-/investigator-dependent (fluorescein angiography, indocyanine green angiography) and involved a qualitative analysis that is not highly reproducible. McClintic et al. [31] was the first to suggest using retinal evaluation for screening CAD in low-risk patients and its adoption as a guideline indication.

The thickness of coronary microvascular structures has been reported to be comparable to that of the retinal arteries, with an independent association between retinal vessel diameters with CAD (wider venules, narrower arterioles, arteriovenous nicking, altered arteriovenous ratio) and of retinal vessel sclerosis with cardiac mortality due to acute coronary syndromes [32,33]. OCT and OCTA go even further and offer the full picture of the retinal microvasculature, not only isolated elements. Individuals exhibiting lower retinal vascular density show inconsistent signs of systemic vascular disease and are more frequently associated with a medical history of peripheral artery disease, CAD, high blood pressure, and type 2 diabetes mellitus. The microvascularization of the retina is closely linked to the cardiovascular risk profile and the severity of coronary lesions [34]. 

In the EYE-MI Pilot Study, Arnould et al. demonstrated a robust association between SCP density and the cardiovascular risk profile, as well as left ventricular ejection fraction impairment in individuals experiencing acute coronary syndromes [35]. On the other hand, Altinkaynak et al. demonstrated reduced subfoveal choroid thickness in patients with heart failure [36]. Ay et al. correlated retinal and optic disk microcirculation alterations with the SYNTAX score: the higher the SYNTAX score values, the lower the ocular microcirculation [11]. On the contrary, Fu et al. examined 57,947 participants with no prior history of coronary artery disease (CAD) from the UK Biobank. Their findings established a noteworthy correlation between retinal microvascular parameters (fractal dimension, number of vascular segments, vascular skeleton density) and the occurrence of CAD. This suggests that a reduced complexity and density of the retinal vascular network may indicate an elevated risk of developing CAD [37]. In addition to diagnostic value, retinal microvascular parameters could hold a high predictive value. 

In 2022, Zhong et al. introduced and validated a nomogram for retinal vasculature, demonstrating its ability to accurately identify the presence of coronary artery disease (CAD). This development aims to enhance patient selection for stress and invasive diagnostic tests, ultimately facilitating a personalized approach based on estimated risk. The model incorporates clinical variables, electrocardiographic signs, and the results of OCTA evaluations. The authors identified three independent OCTA predictors in the retinal vasculature, which include SCP vessel density in the temporal perifovea and nasal perifovea, as well Deep Capillary Plexus (DCP) density in the inferior parafoveal area [11].

Our meta-analysis highlights the potential use of computerized methods such as OCT and OCTA for screening and grading CAD severity. These methods could be easily automated and used for machine-learning assessment of retinal vasculature and integration with electronic health records (risk factors) and coronary evaluation (Gensini and SYNTAX scores) [38] to establish threshold values and stratify risk. This could be of particular interest in evaluating Ischemia with No Obstructive Arteries (INOCA), a highly prevalent entity characterized by microvascular disfunction, ischemia, and no evidence of coronary obstruction at coronary angiography. Finally, retinal investigation could prove a more reliable indicator of the microvascular status than classic cardiac imaging techniques (MRI, CCTA, nuclear tests, contrast echocardiography), especially if the screening protocol takes into account variables such as the time of day in which the evaluation takes place. Chakraborty et al. [39] suggested that potential diurnal variations in ocular biometrics, choroidal thickness, axial length, and intraocular pressure could also impact retinal microvascularization. The main advantages of OCTA are represented by its non-invasive character, fast acquisition times, and easier access by the general population as opposed to standard investigative methods. The question of whether retinal microvascular anomalies are synchronous or preclude coronary lesions remains to be determined. 

There are a series of limitations present in the study that must be acknowledged: (1) heterogeneity was significant (I^2^ > 50%) for most features except for subfoveal choroid thickness, retina thickness, and superficial foveal vessel density. The analysis was performed using the random effect method to consider between-study variance; (2) study quality and publication bias—CAD severity varied across studies, as did the device used for OCT/OCTA and the precise localization of measurements; (3) data were unadjusted for cardiovascular risk factors whose incidence varied across studies (both for CAD and no-CAD patients); (4) we did not analyze all retinal microvascular abnormalities due to a limited number of studies reporting them. 

## 5. Conclusions

The correlation between coronary artery disease and the density and structure of retinal vessels is evident, yet the findings are varied, necessitating further research to create an effective population-based screening and risk assessment tool centered on retinal vascularization.

Retinal vascularization has the potential to serve as a noninvasive biomarker, offering insights beyond conventional routine examinations for evaluating systemic vascular dysfunction.

Diminished vascular density in both the superficial and deep retinal plexuses, along with the thinning of retinal layers, may serve as a valuable subclinical indicator of coronary vasculature impairment, prompting earlier therapeutic and preventive interventions.

## Figures and Tables

**Table 1:** Quality assessment using Newcastle-Ottawa scale.

Criteria	Ahmad M et al. (2017) [13]	Neoh YL et al. (2018) [14]	Wang J et al. (2019) [15]	Kocamaz M et al. (2021) [16]	Zhong P et al. (2022) [11]	Agca FV et al. (2023) [17]	Ay İE et al. (2023) [18]	Matulevičiūtė et al. (2023) [19]	Ren Y et al. (2023) [20]	Sideri AM et al. (2023) [21]	Zhou T et al. (2023) [22]
Selection											
Is the case definition adequate?	★	★	★	★	★	★	★	★	★	★	★
Representativeness of the cases	★	★	★	★	★	★	★	★	★	★	★
Selection of controls	★	★	★	★	★	★	★	★	★	★	★
Definition of controls	★	★	★	★	★	★	★	★	★	★	★
Comparability											
Comparability of cases and controls on the basis of the design or analysis	★★	★	★★	★	★★	★★	★★	★★	★★	★★	★
Exposure											
Ascertainment of exposure	★	★	★	★	★	★	★	★	★	★	★
Same method of ascertainment for cases and controls	★	★	★	★	★	★	★	★	★	★	★
Non-response rate	☆	☆	☆	☆	☆	☆	☆	☆	☆	☆	☆
Total score	8	7	8	7	8	8	8	8	8	8	7

Each criterion can receive a maximum of one star, while comparability can be awarded up to two stars. The maximum score for a study is nine stars.

**Table 2:** Summary of included studies.

Reference	Country	OCT/OCTA Type	Study Type	Methods	Parameters	Patients	CAD Patients (Mean Age ± SD, % of Female Patients)	No CAD Patients (Mean Age ± SD, % of Female Patients)
Ahmad M et al. (2017) [13]	USA	-	Cross-sectional	EDI SD-OCT	Subfoveal CTh and CTh 2000 µm superiorly, inferiorly, nasally, and temporally to the fovea.	62 (single eye)	34 (61.1 ± 6.8, 44.1%)	28 (60.1 ± 5.3, 60.8%)
Neoh YL et al. (2018) [14]	Malaysia	Cirrus	Cross-sectional	Humphrey visual field analysis, OCT	Axial length, ONH disc area, ONH rim area, RNFL thickness, vCDR.	119 (single eye)	59 (59.1 ± 9, 18.6%)	60 (54.1 ± 10.9, 66.7%)
Wang J et al. (2019) [15]	China	Optovue	Cross-sectional	OCTA	Mean retinal thickness, SCP and DCP vessel density, flow area.	316 (both eyes)	158 (66.3 ± 8.4, 54.43%)	158 (64.4 ± 9.2, 55.69%)
Kocamaz M et al. (2021) [16]	Turkey	Heidelberg Spectralis	Cross-sectional	EDI SD-OCT	RNFL thickness, subfoveal CTh and CTh nasally and temporally to the fovea.	85 (both eyes)	53 (61.36 ± 10.57, 18.9%)	32 (57.84 ± 7.52, 18.8%)
Zhong P et al. (2022) [11]	China	Optovue	Cross-sectional	OCTA	RNFL thickness, RPC density, SCP and DCP vascular density, GC-IPL thickness.	410 (single eye)	270 (59.1 ± 9.1, 21.9%)	140 (59.3 ± 6.9, 24.3%)
Agca FV et al. (2023) [17]	Turkey	Optovue	Cross-sectional	OCTA	SCP vessel density whole, DCP vessel density whole, RPC density whole, FAZ area.	185 (single eye)	123 (55.85 ± 7.19, 26.83%)	62 (54.39 ± 6.45, 26%)
Ay İE et al. (2023) [18]	Turkey	Optovue	Cross-sectional	OCTA	SCP vascular density whole, DCP vascular density whole, FAZ area, RNFL thickness.	104 (single eye)	69 (61.5 ± 9, 24.64%)	37 (60 ± 7, 32.43%)
Matulevičiūtė I et al. (2023) [19]	Lithuania	-	Cross-sectional	OCT, OCTA	RNFL thickness, CTh, SCP and DCP vascular density, FAZ area, GC-IPL thickness, retina thickness.	165 (single eye)	92 (59.96 ± 8.44, 36.96%)	73 (59.22 ± 6.95, 45.2%)
Ren Y et al. (2023) [20]	China	Optovue	Cross-sectional	OCTA	RNFL, RPC density, SCP and DCP vessel density.	185 (single eye)	127 (61.57 ± 8.32, 41.73%)	58 (61.91 ± 8.53, 53.4%)
Sideri AM et al. (2023) [21]	Greece	Topcon	Cross-sectional	OCTA	FAZ area, SCP and DCP vascular density, choriocapillaris layer thickness.	330 (both eyes)	88 (55.9 ± 13.7, 6%)	77 (56.6 ± 13.05, 17%)
Zhou T et al. (2023) [22]	China	-	Cross-sectional	OCTA	Choroid thickness.	637 (single eye)	200 (53.43 ± 5.26, 31.12%)	437 (51.41 ± 5.45, 38.5%)

ACS—acute coronary syndrome; CAD—coronary artery disease; CCTA—coronary computed tomography angiography; CTh—choroid thickness; DCP—deep capillary plexus; EDI SD-OCT—enhanced-depth imaging in spectral-domain optical coherence tomography; FAZ—foveal avascular zone; GC-IPL—ganglion cells-inner plexiform layer; IOP—intraocular pressure; MI—myocardial infarction; OCT—optical coherence tomography; OCTA—optical coherence tomography angiography; ONH—optic nerve head; RNFL—retinal nerve fiber layer; RPC—radial peripapillary capillary; SCP—superficial capillary plexus; SD—standard deviation; vCDR—vertical cup-to-disc ratio; VEGF—vascular endothelial growth factor.

**Table 3:** Patient baseline characteristics.

Criteria (Studies Reporting Criteria)	CAD	No-CAD	*p*
Age (weighted mean ± SD) (10 studies)	58.69 ± 9.72	57.73 ± 9.27	0.016
Female patients (no., %) (10 studies)	480 (31.83%)	363 (39.24%)	0.0001
Arterial hypertension (no., %) (11 studies)	874 (56.90%)	220 (48.78%)	0.0023
Hyperlipidemia (no., %) (4 studies)	208 (57.62%)	69 (27.06%)	<0.001
Diabetes mellitus (no., %) (8 studies)	428 (34.91%)	76 (20.11%)	<0.001
Smoking (no., %) (4 studies)	243 (49.89%)	43 (35.83%)	0.0057

SD—standard deviation.

## Data Availability

Data sharing is not applicable.
